# Ethnopharmacological practices by livestock farmers in Uganda: Survey experiences from Mpigi and Gulu districts

**DOI:** 10.1186/1746-4269-10-9

**Published:** 2014-01-27

**Authors:** Immaculate Nabukenya, Chris Rubaire-Akiiki, Deogracious Olila, Kokas Ikwap, Johan Höglund

**Affiliations:** 1 College of Veterinary Medicine, Animal Resources and Biosecurity, Makerere University, P. O. Box 7062, Kampala, Uganda; 2 Department of Biomedical Sciences and Veterinary Public Health, Section for Parasitology, Swedish University of Agricultural Sciences, P. O. Box 7063, Uppsala, Sweden; 3 Department of Biosecurity, Ecosystems and Veterinary Public Health, P. O. Box 7062, Kampala, Uganda

**Keywords:** Ethnopharmacological practices, Herbarium, ITK, Plants, Medicine, Helminthosis, Conservation

## Abstract

**Background:**

There is continued reliance on conventional veterinary drugs including anthelmintics, to some of which resistance has developed. Loss of indigenous technical knowledge (ITK) from societies affects the opportunities for utilization of ethnopharmacological practices unless properly documented. This study was conducted to identify common traditional practices using medicinal plants against helminthosis and other livestock diseases in Mpigi and Gulu districts of Uganda.

**Methods:**

Seven focus group discussions with ten farmers per group plus 18 key informant interviews were held in each district from August to November 2011. Ranking was used to quantify disease burdens and to identify priority livestock and breeds. Samples of each plant were submitted to Makerere University herbarium for identification and documentation. The local name, relative availability and International Union for Conservation of Nature (IUCN) status were recorded.

**Results:**

Seventy six farmers in Mpigi and 74 in Gulu were interviewed. Theileriosis and helminthosis were the most common disease conditions in cattle and goats, respectively. Forty plant species within 34 genera from 22 botanical families were identified, with 20 of these used against helminthosis. Other plants treated wounds and ecto-parasites, theileriosis, retained placenta and bovine ephemeral fever. Non-plant practices (7) and plants cited were used in combination depending on availability. Males older than 40 years had most ethnopharmacological knowledge. Most plants (75%, n = 40) were common, but 10 were rare. IUCN status was not evaluated for 95% of these plants. Conventional and traditional drug use in Gulu and Mpigi districts was different (χ^2^ = 24; p < 0.001). The scientific, English, Luganda and Acholi names of all plants and their availability within the communities are documented herein.

**Conclusion:**

This is the first detailed livestock-related ethnopharmacological study in Gulu district. Farmers in Uganda are still using a variety of practices to treat livestock ailments. Scientific validation and evaluation of conservation status are urgently needed to ensure future availability and knowledge about these plant resources.

## Background

Cattle and goats are the main livestock ruminants kept in rural communities in tropical Africa [1]. In Uganda, more than 80% of the rural population relies on agricultural production, with a varied focus on livestock and crop agriculture depending on the agro-ecological zone [2]. Livestock diseases greatly affect animal welfare, health and productivity, and lead to high treatment costs, losses through reduced growth, unchecked morbidity and mortality [1,3,4]. Helminthosis is among the most debilitating livestock conditions, costing farmers millions of Ugandan shillings through lost production and control efforts [2,5,6]. Notably, gastrointestinal nematodes cause poor weight gains, reduced production, severe weight loss or even direct mortality, especially in small ruminants in resource-poor farming communities [1]. Although many alternative control strategies for helminthosis, such as pasture management [7-9], use of nematophagous fungi [10,11], nutritional supplementation with beneficial forages [12-15], and targeted anthelmintic treatment with the FAMACHA^©^ system, have been put forward [16,17], frequent use of anthelmintics dominates [4], to which helminths have become resistant [18-20].

The World Health Organization estimates that about 80% of the population in developing countries depends on traditional medicine for their primary health care needs [21]. Most of the rural small-holder farmers cannot afford the cost of modern drugs [22], and therefore resort to Indigenous Technical Knowledge (ITK). ITK refers to localized, structured, traditional application of knowledge generated from continuous experimentation and observation of a given phenomenon of interest [23]. In this study, it refers to the use of ethnopharmacological practices, which over a long time have been used to treat diseases and ailments in a given geographical location. The study of ITK is encouraged in Uganda and many African countries because ethnoveterinary practices can supplement modern drugs, and since they are cheaper [24] they are often practiced by farmers [5,22,25]. In addition, drug discovery efforts have been refreshed with the aim of addressing the current resistance problems against the most frequently used anthelmintics [26].

There are a number of studies in Uganda which have documented livestock species-specific ethnopharmacological practices in different parts of the country [25,27-33]. However, due to limited scope, variation of ITK by culture and changing livestock production practices, diversified knowledge from other regions is needed. Before 1986, the Acholi people in Gulu produced a large proportion of cattle and goats, but the district was ravaged by war for more than 20 years. With peace returning, Gulu farmers are currently restocking the land. In contrast, Mpigi district is part of what is known as the cattle corridor, where livestock production is highest in Uganda. Being near the capital city, it is easy to access modern veterinary drugs. With traditional knowledge being replaced by continued use of conventional veterinary drugs, the utilization of indigenous practices is expected to decrease unless properly documented [1]. To document such indigenous knowledge, this study was undertaken to identify the most common practices using plants to treat livestock diseases in Mpigi and Gulu districts of Uganda.

## Materials and methods

### Study design, area and sampling

**Figure 1:**
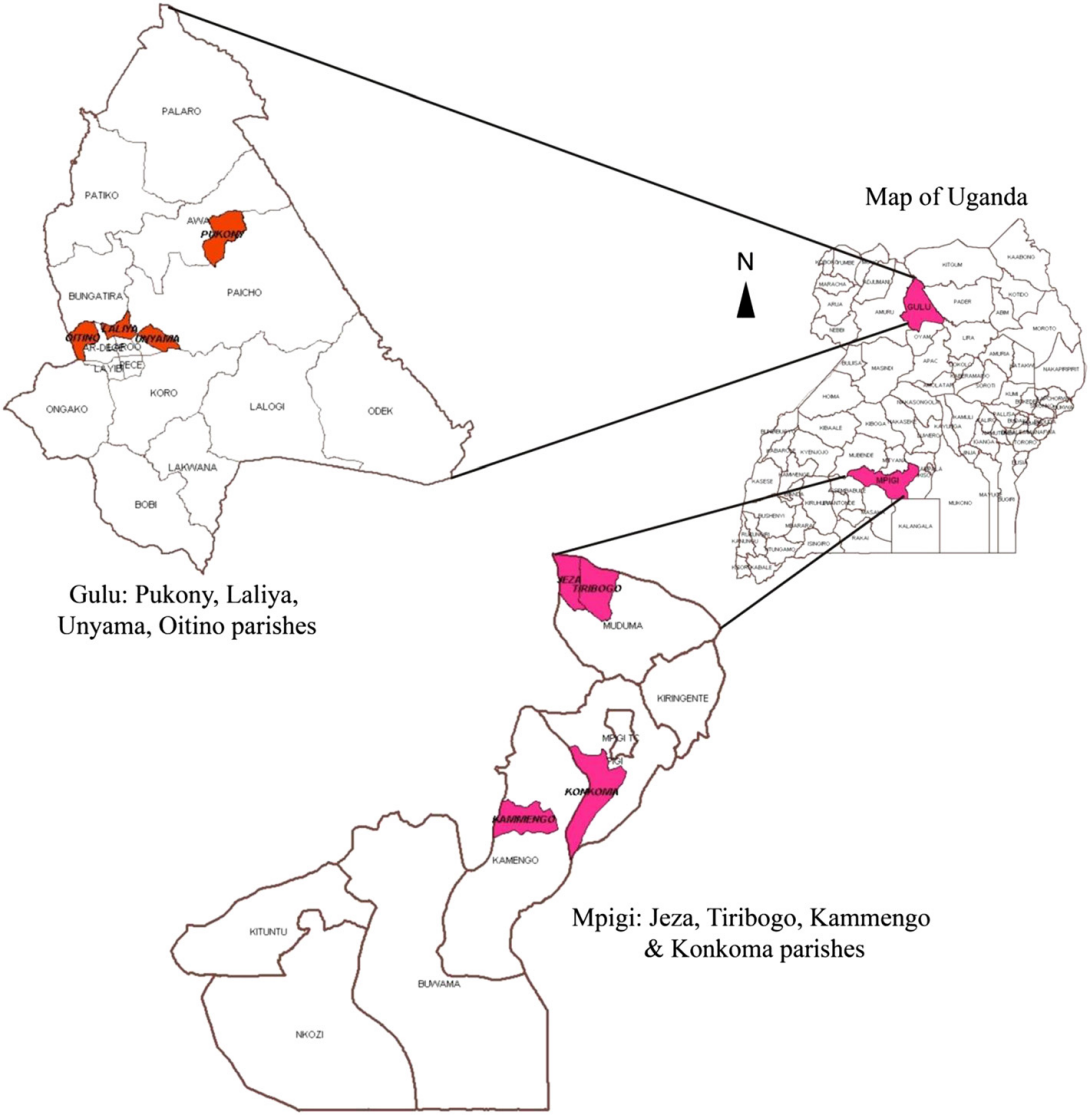
Map of Uganda showing the two study districts, Gulu and Mpigi.

A cross-sectional survey was conducted from August to November 2011 in two districts: Mpigi and Gulu. Mpigi district is located in the central region between 00° 13′ 48″ North and 32° 19′ 48″ East coordinates. The headquarters are located 37 km west of the capital Kampala, and it is part of the intensive livestock/cattle farming corridor in Uganda (Figure 1). Gulu district is located about 340 km north of Kampala, between 02° 45′ North and 32° 00′ East coordinates, with a population that relies on subsistence agriculture [34]. Multistage sampling was used to select the study sites. In each district, two sub-counties were selected. Subsequently, 3 villages in each sub-county were purposively selected [27] with the help of extension staff, making a total of 6 villages in each district.

### Data collection

An assessment of the different ethnopharmacological practices and beliefs among cattle and goat farmers was completed using standard participatory rural appraisal (PRA) techniques [35]. These included focus group discussions (FGDs) with local farmers, key informant interviews (KIIs), scoring and ranking, as well as problem mapping. Visits to collect plant materials and botanical identification were carried out.

### Group discussions

In Mpigi, seven FGDs were conducted in Kammengo, Bwanya, Mpigi Town Council, Bulamu, Konkoma and Jeza villages. In Gulu, seven FGDs were held in Bobi, Unyama, Awach, Purong, Ombachi and Custom Central villages. These villages were selected because they represented the peri-urban and rural agricultural production systems. Seventy six and 74 farmers were interviewed in Mpigi and Gulu, respectively, with an average of 10 people (both male and female) per group. Participants were livestock farmers owning at least five ruminants and with more than five years of livestock farming experience. A standard interview guide was used to ensure uniformity of the data collected during the FGDs, after the participants had signed a consent form. The discussions were taped and kept in the local languages (Luganda and Acholi) but later translated for the researchers.

### Interviews

In each district, nine KIIs were held, four with veterinary extension workers and five with community ITK persons, using a semi-structured interview guide. Both in the KIIs and FGDs information was gathered on priority livestock and prevailing diseases including helminthosis. Perceived causes, disease diagnosis, treatment methods, plant sources, herbal preparations and dosages were documented. The conservation efforts and challenges encountered while using herbal medicine were also documented.

### Matrix scoring and ranking

To understand how diseases were diagnosed and treated, participants were involved in practical scoring and ranking of diseases and their treatment. The participants were provided with 100 bean seeds each, which they used to indicate differences in availability of the plant species or varieties mentioned, and changes in burden of disease with livestock species and general priorities. This helped to quantify some items in the checklists and achieve agreement on a given variable within the group.

### Plant identification

A local name was attached to each plant, and information on its relative availability was recorded [31]. Reference samples of each plant were submitted to Makerere University herbarium for identification by a botanist, and voucher specimens were preserved. Review of each plant’s conservation status data was done in reference International Union for Conservation of Nature (IUCN) red list (http://www.iucnredlist.org) and Convention on International Trade in Endangered Species (CITES) classification (http://www.cites.org).

### Data management and analysis

The data were transcribed and entered into Microsoft Excel 3.0. During analysis, data were summarized into major themes by content analysis [35]. Descriptive statistics were obtained for quantifiable data in Statistical Package for Social Scientists (SPSS version 15.0). The scientific family and species names of the plants were obtained after botanical identification.

## Results

### Socio-demographics of respondents

A total of 150 participants were interviewed in seven FGDs conducted in each district. Of these, 87 (58%) were female and 63 (42%) male. On average, the farmers interviewed were 36 ± 9.8 years old (mean ± standard deviation). Whereas 15 (10%) participants had no formal education, 83 (55%) had primary and 62 (35%) secondary education level. The average number of goats was 8.6 ± 3.9 with a minimum of 2 and maximum of 56 goats. The average cattle number was 3.2 ± 2.9 with a maximum of 49. Twenty percent (n = 150) of the farmers had only goats. The average household land size was 2.5 ± 2.9 acres (range 1–30) in Mpigi and 6.5 ± 3.5 acres (range 3–50) in Gulu district.

### Major livestock species kept

FGDs in Mpigi revealed that the priority livestock was local breeds of cattle followed by chicken (Table 1). Most (>70%) of the pigs, goats and sheep were local breeds, with few crosses and exotic breeds. Species ranked highly in Gulu were goats followed by cattle. Most of the chicken, cattle and goats were local breeds, whereas pigs were exclusively exotic or cross breeds (Table 1).

**Table 1:** Priority livestock species and common breeds in Mpigi and Gulu districts

			**Breeds**			**Management system**		
**District**	**Livestock species**	**Priority %**	**Local %**	**Crosses %**	**Exotic %**	**Free range %**	Semi-intensive* %	**Intensive %**
**Mpigi**	Cattle	35	47	48	5	15	80	5
	Chicken	30	47	3	50	50	0	50
	Pigs	25	80	17	3	0	80	20
	Goats & sheep	10	85	13	2	5	90	5
**Gulu**	Goats**	50	80	15	5	20	78	2
	Cattle	30	80	15	5	90	5	5
	Pigs	10	5	50	45	10	20	70
	Chicken	10	90	2	3	90	0	10

*Semi-intensive management method is tethering (tying animals with a rope at a different fixed grazing/feeding area each day).

**Sheep are much less common; preference for goats overrides.

### Prevalent livestock diseases and conditions

Theileriosis or East coast fever (ECF), known as “Amakebe” in Luganda and “Oding ding” in Acholi, was ranked by the farmers as the most common cattle disease in both districts (Table 2). Helminthosis was a commonly encountered condition in all priority species. It was ranked number one in goats and sheep, second in pigs, but was less important in chicken (Table 2). Descriptions of the most common diseases and conditions during interviews were usually accurate. Notably, ECF was not well understood, with farmers claiming that it mostly affects young cattle whose lymph nodes swell when they consume a lot of milk. Helminthosis was well described, including clinical signs such as emaciation and weight loss, rough hair coat, reduced appetite, “pot belly” and detection of worms or segments in faecal matter. Apart from farmers with exotic livestock and chicken breeds, 80% of the local farmers did not regularly treat against helminthosis. However, they treated affected animals, especially when some had diarrhoea or died.

**Table 2:** Most prevalent livestock and chicken diseases in Mpigi and Gulu districts

**Animal species affected**	**Disease name**	**Local name - Luganda**	**Local name – Acholi**	**Rank (1-Highest prevalence - Mpigi)**	**Rank (1-Highest prevalence – Gulu)**
Cattle	East coast fever	Amakebe	Oding ding	1	1
	Coughing	Okukolola	Aona	2	6
	Helminthosis	Enjoka	Kwidi, odini (liver flukes)	3	2
	Heart water	Mulalama	?	4	9
	Mange	Olukuku	Angoli	5	7
	Trypanosomosis	Kipumpuli	Tu o jonyo	6	3
	Bovine ephemeral fever	Kamenyo	Okwero	7	10
	Bloat	Kamukuulo	Deng ici	8	5
	Ticks & biting flies	Enkwa & Ebiwuka	Okwodo & Lwangu	9	4
	Mastitis	Ebbani	Angoli me tunu	10	8
Goats and Sheep	Helminthosis	Enjoka	Kwidi	1	1
	Mange	Olukuku	Angoli	2	3
	Heart water	Mulalama	Awila wic	3	4
	Parapox or orf	Obumwamwa	Abworu	4	2
	Abscesses	Ebizimba	Buu	5	5
Pigs	African swine fever	Omusujja gw’embizzi	Orere pa opego	1	1
	Helminthosis	Enjoka	Kwidi	2	2
	Swollen udders	Okuzimba amabeere	Cak pa dyang/dyel ma kwot	4	5
	Mange	Olukuku	Angoli	3	4
	Lice	Ensekere	Nyugi	5	3
Chicken	New castle	Kiwumpuli	Orere pa gwenu	1	1
	Coccidiosis	Kiddukano	Orere	2	2
	Helminthosis	Enjoka	Kwidi	5	4
	Mites and fleas	Obuloolo, enkukunyi	Ladep, Lakuny	3	5
	Flu	Senyiga	Abworo	4	3

### Treatment of animal diseases

**Figure 2:**
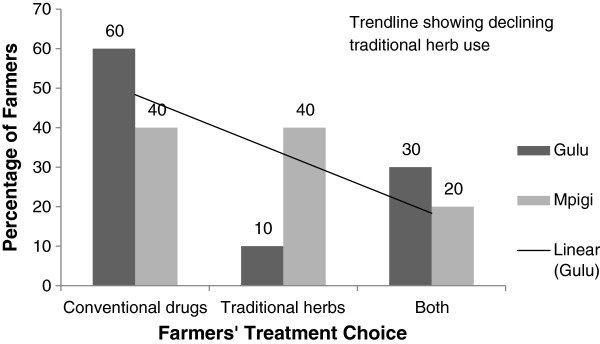
Farmers’ choice and practices for animal disease management in Gulu and Mpigi districts.

Treatment depended on availability of funds to procure the conventional drugs (49%), availability of veterinary services (22%), knowledge of alternatives such as ITK (15%), cost attached to the animal (10%) and seriousness of the condition (6%). Owing to low availability of service providers, 55% of the farmers treated their own animals. The majority (60%) of the farmers used the conventional drugs to treat their animals especially cattle, pigs and chicken (Figure 2). Generally during discussions, participants aged below 40 years showed less knowledge about alternative treatment options and ITK. This was more pronounced in Gulu than in Mpigi district where the different plant names and their uses were discussed by farmers older than 40 years. Relatedly, 25% of the farmers with knowledge relied entirely on the alternative herbal preparations to treat animal diseases (Figure 2), as demonstrated in this excerpt:

*“We used the plants before, but we learnt over time that when you want maximum returns or when your animals are crosses and exotic breeds, the choice is to use veterinary drugs, thus we changed”* - Female FGD Respondent, Mpigi district.

The reasons for low level of use among the knowledgeable farmers were: increased trust in conventional drugs (60%), scarcity of the plant resources (30%) and lower demand from other farmers (10%). There was a significant difference between conventional and traditional drug use in Gulu and Mpigi districts (χ^2^ = 24; p < 0.001). Farmers in Mpigi mentioned six plants (no Acholi names specified in Table 3), which were not used in Gulu. The latter mentioned one plant, *Hyparrhenia rufa* (“Ajuu”) not used in Mpigi. Different plant names in English, Luganda and Acholi were given (Table 3).

**Table 3:** Plants used to treat different diseases/conditions and how they are used

Relative availability*; IUCN status^**a**^	**Voucher number**	**Family**	Species and common name***	**Vernacular name (*Luganda, Acholi *)**	Habit; part used****	**Preparation method (Amounts vary with species and age of animal)**
**Disease/condition: Helminthosis in all livestock and poultry**						
Common weed, invasive: +++; NE	NI 039	Euphorbiaceae	*Euphorbia heterophylla* Linn. Klotzsch & Garcke (Milk weed)	Kisandasanda	H; L	- Boil leaves and drench (too much causes diarrhoea)
Rare weed by road sides: +; NE	NI 002	Caesalpinaceae	*Senna occidentalis* (L.) Link (Stinking weed)	Muttanjoka; Ayila	S; L, R	- Pound roots, add water and rock salt then drench
						- Crush fresh leaves, or use dried powder, add water and drench
Ubiquitous, easily located: ++; NE	NI 003	Asteraceae	*Vernonia amygdalina* Delile Kuntze^a^ (Bitter leaf)	Mululuza; Labwori	S; L, R, B	- Crush fresh leaves, add water and drench
						- Boil roots with water and give to drink
						- Give animal that has just delivered fresh leaves to eat
Rare due to restrictions: +; NE	NI 036	Cannabaceae	*Cannabis sativa* (Lam.) E. Small & Cronquist ^*b*^ (Hemp, Marijuana)	Njaga; Lakera	H; L, S	- Crush fresh leaves or add dried powder and mix with water
						- Crush, mix with water and crude lake salt and give to drink
Wild, some grow it: ++; NE; APP II*	NI 038	Xanthorrhoeaceae	*Aloe vera* (L.) Burm. f (Babados aloe)	Kigaji	H; L	- Slice and boil the leaves and give to drink
						- Slice fresh leaves and add to feeds
Commonly grown for sale: +++; NE	NI 021	Solanaceae	*Nicotiana tabacum L.* (Tobacco)^c,d^	Taaba; Muvuavui	H; L	- Crush leaves and mix with water then drench
						- Boil dried leaves, leave to cool and drench
Commonly eaten in households: +++; NE	NI 040	Solanaceae	*Capsicum annuum* (Dunal) Heiser & Pickersgill	Kamulali; Obolo	H; L, Fr	- Crush fruits or leaves, mix with ash and water then drench
						- Crush fruits or leaves and mix with tobacco or cannabis and ash then drench
Common fruit: +++; VU	NI 037	Caricaceae	*Carica papaya L.* Linn. (Pawpaw)	Papaali; Owak	T; R, S	- Roots crushed, boiled with water and little paraffin, then drench
						- Dried seeds crushed and boiled with water, then drench
Ubiquitous: ++; NE	NI 005	Lamiaceae	*Tetradenia riparia* (Hochst.) Codd (Ginger-bush)	Kyewamala; Omwombyer	S; L	- Crush leaves, mix with water and give to drink - Crush leaves and put on the wound
						- Drench with decoction before calf suckles (ECF)
Ubiquitous by roadsides: +++; NE	NI 001	Caesalpinaceae	*Senna hirsuta* (L.) H. S. Irwin & Barneby (Stinking cassia)	Gasia; Coga macon	S; L	- Boil dry leaves with little rock salt and drench or crush fresh leaves and drench.
						- Cut and give the animals as fodder
Wildly growing: ++; NE	NI 004	Leguminosae	*Tephrosia vogelii* Hook f. (Fish-poison bean)	Muluku; Kineke	S; L	- Crush fresh leaves and drench
						- Boil dried leaves, cool and drench
Cultivated: ++; NE	NI 032	Euphorbiaceae	*Jatropha curcas* Linn. McVaugh (Purging nut)	Kiloowa; Olwiro	S; L, Fr	- Crush fresh leaves and fruits then drench
						- Crush leaves and put on the wound
Wildly growing: ++; NE	NI 033	Euphorbiaceae	*Ricinus communis* Linn*.* (Castor oil)	Nsogasoga; Laliya	S; L, S	- Decoction by boiling leaves and crushed seeds; cooling and drenching
Wildy growing or planted: ++; NE	NI 006	Meliaceae	*Azadirachta indica* Linn*.* A. Juss. (Neem tree)	Niimu; Nyakanyaka	T; L	- Crush fresh leaves and drench
						- Crush fruits, boil them, cool and drench
Rare herb: +; NE	NI 031	Leguminosae –Papilionoideae	*Pseudarthria confertiflora* (A. Rich.) Baker	Kikakala	H; L, B	- Boil leaves or bark for one hour, cool and drench
Common weed, invasive: +++; NE	NI 027	Poaceae	*Digitaria abyssinica* (A. Rich.) Stapf (Couch grass)	Lumbugu; Coga macon	G; L, R	- Boil leaves and roots, cool then drench
Wildly growing, rare: +; NE	NI 034	Caesalpinaceae	*Senna didymobotrya* (Fresen.) H. S. Irwin & Barneby (Peanut butter cassia)	Mukyula; Lakera/Lurogo	T; L	- Boil leaves, mixed with *Senna occidentalis* and drench
						- Crush fresh leaves, mix with water and spray on skin (ectoparasites)
Grown in some homes: ++; NE	NI 007	Moringaceae	*Moringa oleifera* Lam. (Horseradish tree)	Moringa	S; L, Fr	- Crush fresh leaves, add ash and red pepper then drench
						- Boil dried leaves then cool and drench
Wildly growing: ++; NE	NI 026	Papilionoideae	*Erythrina abyssinica* Lam. ex DC.^*e*^ (Flame tree)	Jirikiti; Lucoro	T; B	- Pound the bark and leave to dry. Soak 3 handfuls of pounded dried bark in water (2 hours) and drench 1 cow or 2 goats.
Wildly growing: ++; NE	NI 028	Phytolaccaceae	*Phytolacca dodecandra* L'Her. (Endod)	Luwoko; Olango	H; L, Fr	- Infusion of the leaves, fruits then drench
**Disease/ condition – African Swine Fever**						
Practice 1				Human urine	N/A	- Collect, add ash and ethanol, give orally
**Disease/ condition - New Castle Disease**						
Practice 2				Human urine	N/A	- Mix with ash and rock salt, give to drink
In homes: ++; NE	NI 008	Solanaceae	*Capsicum frutescens* L. Kuntze (Red pepper)	Kamulali; Pilipili	H; L, Fr	- Add ash + water to freshly squeezed leaves then drench
						- Mix with *Cannabis sativa*, sisal (*Agave sisalana*) juice and ash then drench
						- Mix with *Aloe spp (Flower and leaf)+* Opium.
**Disease/condition: Bovine Ephemeral Fever**						
Wildly growing: ++; NE	NI 022	Acanthaceae	*Acanthus pubescens* (Thomson ex Oliv.) Engl. (Grey goddess)	Amatovu; Achika	S; L	- Beat the legs with ends of the leaves until animal stands
Wildly growing: +; NE	NI 016	Vitaceae	*Cyphostemma adenocaule* Descoings. ex Wild & R. B. Drumm. (None specified)	Kibombo; Ogali	H; L	- Crush leaves, add ash and drench
In grazing lands: ++; NE	NI 018	Asteraceae	*Albizia anthelmintica* Brongn (Cherry-blossom tree)	Mweramanyo; Owak	T; L, B	- Decoction from leaves and bark then drench
Common weed: ++; NE	NI 029	Lamiaceae	*Leonotis nepetifolia* (L.) R. Br. (Sun-bird flower)	Kifumufumu; Okwero/ Achika	H; L	- Drench with warm decoction twice daily for three days
Practice 3					N/A	-Drench with decoction from boiled grass hoppers
**Disease/condition: Constipation**						
Rare herb: +; NE	NI 035	Cucurbitaceae	*Lagenaria sphaerica* (Sond.) Naudin (Wild calabash)	Kifuula; Lango	H; L	- Drench of decoction made by boiling leaves, adding little rock salt and cow ghee
Practice 4				Cooking oil	N/A	Use large bore tube e.g. horse pipe to give orally
Practice 5				Omo liquid/very soapy water	N/A	Give animal to drink
**Disease/condition: Theileriosis/ East Coast Fever**						
In grazing lands: +; NE, APP II*	NI 009	Euphorbiaceae	*Euphorbia candelabrum* Tremaux ex Kotschy (Toothbrush tree)	Enkukuulu; Acak-Acak	T; La	- Break and drop sap onto the lymph node or wound
						- Decoction drench removes afterbirth
Around paddocks: ++; LC	NI 010	Euphorbiaceae	*Euphorbia tirucalli* L. Klotzsch & Garcke (Pencil tree)	Lukoni, Labuka	S; La	- Break the leaves and put sap on the lymph nodes or wounds
Rare, in grazing lands: +; NE	NI 025	Lamiaceae	*Clerodendrum myricoides* (Hochst.) Moldenke Butterfly weed	Mukuzanyana; Okwero	S; R	- Pounded roots put in boiling water for one hour, cooled then drench
						- Same to treat Bovine Ephemeral Fever
						- Same for helminthosis
Wildly growing: ++; NE	NI 030	Asteraceae	*Microglossa pyrifolia* (Lam.) Kuntze (Secondary bush)	Akafugankande	S; L, R	- Decoction of the roots then drench to relieve respiratory distress in ECF
Wildly growing: +; NE	NI 012	Euphorbiaceae	*Euphorbia umbellata* (Pax) Bruyns (African milk bush)	Kafumba	H; L, Fl	- Break leaf and put sap on lymph node; irritate and dry the skin
**Condition - Lice, fleas, mites, mange and wounds**						
Commonly grown: +++; NE	NI 020	Poaceae	*Sorghum bicolor* (L.) Moench (Sorghum) - Fermentation residue	Muwemba; Any wagi - Enkanja	G; S	- Mix crushed fruits with roots of *Cyphostemma adenocaule* and apply on wound
						- Smear on the skin
Wildly growing: ++; NE	NI 023	Cucurbitaceae	*Momordica foetida* Schumach. (Snake food)	Bombo; Bomo	H; L	- Water extraction of the leaves, drench
Wildly growing: ++; NE	NI 015	Lamiaceae	*Hoslundia opposita* Vahl (White-tipped hemizygia)	Kamunye; Odwongo	S; L, Fl	- Decoction applied daily externally with pressure on the wound for a week or more
Practice 6				Methylated Spirit	N/A	- Apply topically on wounds
Practice 7				Soapy water		- Add OMO^R^ and wash the skin
**Disease/condition: Parturition failure**						
Grown food: +++; NE	NI 013	Convolvulaceae	*Ipomoea batatas* (L.) Lam (Sweet potato)	Amalagala; Maku	H; L	- Give animal the vines to eat
						- Crush leaves without water and smear on the vulva
**Disease/condition: Retained placenta**						
Grazing land weed: ++; NE	NI 014	Poaceae	*Hyparrhenia rufa* (Nees) Stapf (Jaragua grass)	Ajuu	G; R	- Remove roots from stump, boil for one hour, cool and drench animal
Wildly growing: ++; NE	NI 019	Anacardiaceae	*Rhus natalensis* Bernh. ex Krauss (Red cape beech)	Kakwansokwanso; Atakarach	H; L; Fr	- Fresh fruits and leaves are crushed and mixed with water, sieved and drenched
Rare, in forests: +; NE	NI 024	Ebeneceae	*Diospyros abyssinica* (Hiern) F. White (African ebony)	Mpojwa	T; L, Fr	- Leaves and dried fruits are crushed and a decoction is drenched
Ubiquitous wildly growing: +++; NE	NI 011	Solanaceae	*Solanum incanum* (L.) Kuntze (Sodom apple)^*f*^	Entengotengo; Ocuga	H; L, Fr	- Smear crushed fresh fruit around the vulva
						- Squeeze sap from burnt fruit to wound
Wildly growing: ++; NE	NI 017	Solanaceae	*Solanum aculeastrum* Dunal (C. H. Wright) Bitter (Thorny apple)	Kikutizangalabi	S; Fr	- Squeeze fruits in water or milk then given orally as drench (cough)
						- Heat ripe fruits and smear on the vulva

*Availability of the plants varies; + Rare or endangered; ++ Available but not common; +++ Ubiquitous and quite common.

**Month and year indicated for collection date.

***One commonly used English name; some have many while others have none as indicated in the table.

****Habit: G: Grass; H: herb; Li: Liana; S: shrub; T: tree.

**Plant parts:** B - Bark; L - Leaves; La - Latex; Fl - Flowers; Fr - Fruits; R - Roots; S - Seeds.

^a^Boil leaves or crush fresh leaves then drench; also used for stomach ache and fever in People or animals.

^*b*^Used on wounds, spray to prevent mites.

^c^Crush and mix with water then spray animal to treat ectoparasites.

^d^Crush and boil leaves – drench treats uterine prolapse; decoction also treats cattle skin diseases including mange.

^e^Also for estrus induction (bring animal to heat) and smear on the wounds for wound healing.

^f^Also treats theileriosis and wounds.

Legend Table 3:

A summary of 40 plants and seven practices used in treatment of livestock and chicken diseases in Gulu and Mpigi districts. Details of preparation, plant part used, conservation/biodiversity status, common English, Acholi and Luganda names are provided. Twenty plants were used to treat helminthosis; wounds and ectoparasites (8), Theileriosis (6), retained placenta (5), Bovine Ephemeral Fever (4), and one each for Newcastle disease, uterine prolapse, constipation and retained placenta.

### Indigenous technical knowledge used

This study established that 40 plants from 34 genera and 22 families are used to treat different diseases and conditions. *Euphorbiaceae* (15%) followed by *Solanaceae* (13%) were the commonest plant families. Twenty plants were used to treat helminthosis, wounds and ecto-parasites (8), theileriosis (6), retained placenta (5), bovine ephemeral fever (4), and one each for Newcastle disease, uterine prolapse, constipation and retained placenta. Some plants were indicated for more than one disease/condition. The resource persons (90%) acquired the ITK through inter- and intra-generation oral tradition within the family. Most plants were used in fresh form. A few rare plants (10%), tree leaves and roots were preserved in dried powder or whole form and constituted when needed. The oral route (80%) for systemic conditions and topical application (15%) for ecto-parasite control were the most common modes of administration (Table 3). Leaves (75%) bark (15%) and roots (5%) were most commonly used plant parts. Farmers knew the danger of relying on roots as the source of active components, as shown in this excerpt:

*“We only use chips of roots from big trees so that we do not completely destroy the source. Most of these plants are rare and many people use the same tree; thus we do not encourage use of roots in traditional medicine”,* Female FGD Respondent, Gulu district.

Seven non-plant practices such as orally administering cooking oil and soapy water to treat constipation were cited. Human urine mixed with ash and a decoction of Ugandan edible grasshoppers were also used (Table 3).

### Perception of efficacy and risks of medicinal plants

The majority (80%) of farmers with knowledge of ITK said and believed the practices and plants were efficacious and safe, showing minimal side effects. Doses therefore were not standardized but indicated roughly. For example, a *“mound”* or *“handful”* of plant material mixed with a *“bottleful”* of water was more common expressions than quantifiable amounts or volumes. However, they said that some plants, such as *Phytolacca dodecandra* and *Senna occidentalis*, if used in large doses posed serious toxicological effects.

### Sources of herbal medicine

**Figure 3:**
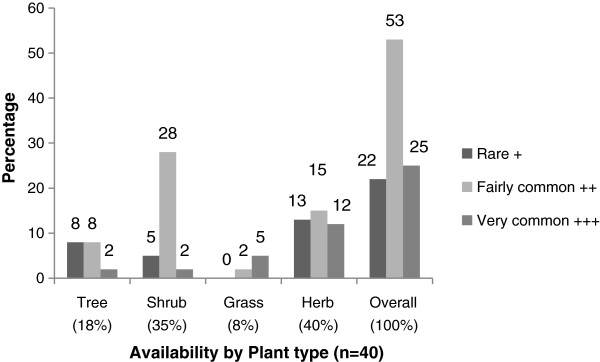
Relative availability of plant materials within communities.

Farmers obtained > 80% of the plants from wild flora or weeds, usually near their homes. About 15% of the shrubs or herbs were specifically grown or conserved around homes for medicinal purposes. The longest distance traveled for rare plant materials was 15 km in Gulu district. The majority (>70%) of shrubs and forest trees were obtained within 2 – 6 km. The access distances were shorter in Mpigi district and the longest distance was 5 km from designated forests. Herbs followed by shrubs were the most common plant types (Figure 3). A graph of relative availability of the plants demonstrated that 53% were fairly common; mostly the shrubs and 12% of the herbs were very common. Trees were rare or fairly common, depending on the species (Figure 3). International conservation status of 95% of plants was not evaluated (NE) by IUCN except *Euphorbia tirucalli* L. which was categorised as least concern (LC) and *Carica papaya* L. as vulnerable (VU). Categorisation by CITES indicated family Euphorbiacaea and *Aloe vera* as plants traded with caution (Table 3).

### Challenges

Several challenges in using traditional knowledge and herbal medicine in treatment of livestock diseases were mentioned. The top three ranked were:

1. Most of the plants were used in combination, and limited access to some rare ones prohibited the full scale use of such ITK.

2. Most treatments were not openly shared, with few people having the knowledge.

3. The doses were not well standardized, with wide variations in treatment duration and amounts to administer.

## Discussion

This is the first detailed survey of livestock ethnopharmacological practices from Gulu district. Evidently, there was lower utilization of ethnopharmacological practices in Gulu district compared to Mpigi. In Gulu district, younger farmers (<40 years) portrayed less knowledge, possibly as a result of the breakdown in the ‘traditional social safety nets’ breakdown as an outcome of war or insurgencies in the district [36]. The role of traditional social safety nets adaptation and resilience has been recognized in Zimbabwe [37]. Similarly, goats instead of cattle were first priority due to reduced land sizes and cattle losses incurred during the previous war. The impact of the war is still felt in the economic performance of the district [38,39].

The current study established 40 plants from 34 genera and 22 families, and 7 non-plant practices, used to treat livestock diseases. A previous global study documented that more than 119 conventional drugs prescribed were derived from plants. The bioactivity of 74% of 119 plant-derived drugs was discovered during validation studies of documented traditional uses of the respective plants [40]. Therefore, the use of the plants identified in the present study in advanced searches for new drugs is paramount and can lead to novel discoveries. Documentation of ITK nevertheless, guards against loss of traditional knowledge due to limited inter-generation transfer [24,25].

More females than males participated, and a majority of the respondents at least had primary level education. Women have better health seeking behaviour than men, and herbal medicine is practiced in a similar pattern [21,27]. According to this study, men mainly did the ethno-diagnosis and treatment of livestock diseases. This is probably because taking care of large livestock such as cattle is by tradition considered male gender role in Uganda. This could also mean that much of ITK in livestock is mainly passed to the next generation through men. The participation of more women than men during FGDs could therefore mean that women considered this an opportunity to learn more about livestock. Indeed, FGDs in this study were a learning platform for many participants, who exchanged information on the different plants used for treating different diseases and conditions in livestock.

The numbers of plants and preparation methods described in this study were restricted to the prioritised diseases/conditions for the animal species identified (Table 2). In addition, the study documented the scientific, English, Luganda and Acholi names, which enhances intra- and inter-generation dissemination. The relative availability and conservation status of the different plants was reported, which few studies have achieved.

The most common mode of preparation was water extraction (75%), where the plant parts were crushed and mixed with water before drenching. This is similar to previous findings [27] that showed the use of 37 plants against helminthosis in livestock from pastoral communities in Uganda. Decoction (boiling plant parts in water) was more common than infusion (submerging plant parts in hot water), and this is in agreement with a previous report [25]. However, this is contrary to a finding that most plant preparations in Bulamogi county, Uganda, were by infusion and less commonly by decoction [32]. The study in Bulamogi described more than 200 plants and practices for treatment of various human ailments but with less focus on livestock diseases.

Different studies have documented usage of some of these plant species in different parts of Uganda and Kenya [5,25,27,28,30,32,41,42]. All of these studies are area-specific, prospecting for different livestock or poultry diseases, whereas this study was focused on the farmer prioritized conditions and remedies. In one study, up to 29 plants were documented for the treatment of ECF, five for diarrhea and one against intestinal worms [32]. Another study [27] documented 11 of the plants mentioned in the current study but five of these were prepared differently. Such variations in methods may cause significant difference in bioactivity of plant material due to variation in concentration of bioactive compounds [3] even when the same plant is used. In a study of household herbal medicine used in four districts of Uganda, 41 plants were documented but noted the loss of ITK [31]. In pastoral Karamoja, 209 plants treating 130 conditions were documented [25]. A study in Mpigi district in 1993 documented 46 plants used for a wide range of conditions [5] but only 16 (35%) of these were reported in the current study. What is interesting is the wide variation of ITK over time, even in the same or similar geographical locations.

Knowledge levels decreased by age, and the people younger than 40 years were less knowledgeable, which is similar to findings in Kenya [42]. Also similar to previous findings [5,42], males in the present study were more knowledgeable than females of similar ages. The information was not freely shared, unlike in pastoral areas [3,25], possibly because some people were believed to have native treating ability. The farmers believed most plants were safe and non-toxic [25,31]. However, plants known to be toxic were used, albeit with caution in advance. In these cases, there was an attempt to give clear amounts within which the doses were safe. Use of non-plant practices, like cooking oil and soap, has been documented before [27,30,42]. It is notable that these practices varied with the condition and usually no standard amounts were specified. The use of plants and non-plant practices in this study demonstrated that farmers have great wealth of knowledge of disease conditions affecting their animals. The validation of this information through well designed scientific research and dissemination of such findings will enhance ITK utilization.

## Conclusion and recommendations

In Mpigi and Gulu districts, farmers used a variety of 40 plants and seven non-plant practices to treat livestock and poultry diseases. More than half of these plants were readily available in their environment while 25% were rare, and thus their use is compromised. The ethnoveterinary knowledge level was low among people below 40 years, especially in Gulu which may be attributed to the negative impact of prolonged war on the traditional social safety nets which are crucial in imparting ITK to the young folks. This was exhibited by fewer farmers practicing ITK in Gulu District. This calls for heightened sensitization about use and conservation of these plant resources in this district. Validation of efficacy of these ethnopharmacological products is also imperative to enhance full-scale use of these products in livestock production.

## Abbreviations

ECF: East coast fever; FGDs: Focus group discussions; ITK: Indigenous technical knowledge; KIIs: Key informant interviews.

## Competing interests

The authors declare that they have no conflict of interest.

## Authors’ contributions

All authors contributed equally to this work. IN and KI took the lead in design and field implementation of the study, whereas JH, RA, and DO supervised the work and supported the writing process. JH, RA and IN contributed to data analysis and presentation. RA, KI and JH supported the editing process. All authors read and approved the final manuscript.
